# Supplementary material for: The Food Bank and Food Pantries Help Food Insecure Participants Maintain Fruit and Vegetable Intake During COVID-19
Source: Front Nutr. 2021 Aug 6;8:673158. doi: 10.3389/fnut.2021.673158 (PMC8378669; doi:10.3389/fnut.2021.673158)
Supplement: Supplementary file 1 [file Data_Sheet_1.docx]

Supplementary Material

# Supplementary Figures and Tables

**1.1 Supplementary Table 1.** Use of dependent and independent variables in statistical analysis.

| Variable | Measurement | Scale |
| --- | --- | --- |
| Food (In)Secure | 6 item food security module from USDA. Statements shown in Figure 2. | Binary variable (Affirmative to 2 or more questions= Food Insecure), 0 or 1 affirmatives= Food Secure |
| Fruit Intake | About how many cups of fruit (including 100% pure fruit juice) do you eat or drink each day? Examples of 1 cup for fruit include 1 small apple, 1 large banana, 1 cup (8 oz.) of 100% juice or canned fruit, or ½ cup of dried fruit. (Yaroch et al. 2012) | 0= None, 1= ½ cup or less, 2= ½ to 1 cup, 3=1–2 cups, 4= 2–3 cups, 5= 3–4 cups, 6= 4 cups or more |
| Vegetable Intake | About how many cups of vegetables (including 100% vegetable juice) do you eat or drink each day? Examples of 1 cup of vegetables include 1 cup of cooked leafy greens, 2 cups of lettuce or raw greens, 12 baby carrots, 1 medium potato, or 1 large raw tomato. (Yaroch et al. 2012) |  |
| Fruit/Vegetable Change | I have been eating more, less, or about the same amount of fruits and vegetables per day. | 1= Less, 2= Same, 3= More |
| Food Pantry Use | Which of the following food assistance programs did your household use in the year before the COVID-19 outbreak, if any, and since the COVID-19 outbreak (March 11)? | 1= Use of "food pantry/food bank" since COVID-19 outbreak, 0= No use of "food pantry/food bank" since COVID-19 outbreak. |
| Shared or Public Transportation Use | What were the typical types of transportation you used to get food for your households, in the year before the COVID-19 outbreak and since the COVID-19 outbreak? Check all that apply | 1= use of bus or other public transit, ride from friend/family/neighbor, ride from taxi, someone brings food to me, walk or bike since the COVID-19 outbreak. 0= no use of these modes |
| Female | Which of the following best describes your gender identity? | 1= Female, 0=Male |
| Children in HH | How many people in the following age groups currently live in your household (including you)? Household includes people currently living within your home, including family and non-family members. | 1= Any children in household, 0= No children in household |
| Over 55 | Please select your age group | 1= Respondent 55 or older, 0= Respondent 55 or younger |
| Race/Ethnicity (BIPOC/Hispanic) | What is your race? Are you of Hispanic, Latino, or Spanish origin? | 1= Respondent identify as Asian, Black or African America, Native America, White, Mixed Race, and/or Hispanic, Latino or Spanish origin, 0= Respondent identifies as white and non-Hispanic, Latino or Spanish origin |
| Any Job Change | Have you or anyone in your household experienced a loss of income or job since the COVID-19 outbreak (March 11)? | 1= Any job change (job loss, reduced hours or income at job, furloughed), 0= No job change |
| Less $50K | Which of the following best describes your household income range in 2019 before taxes? | 1= Household income below $50,000 a year, 0= Household income above $50,000 a year |
| HH Size | How many people in the following age groups currently live in your household (including you)? Household includes people currently living within your home, including family and non-family members. | 1= 1 person, 2= 2 people, 3= 3 people, 4= 4 people, 5= 5 people, 6= 6 people, 7=7 people or more |

**1.2 Supplementary Table 2.** Food insecurity by disaggregated race and ethnicity.

|  | Food Insecurity Rate | |  |  |
| --- | --- | --- | --- | --- |
|  | For Demographic Group | For Outside Demographic Group | Total Number of Respondents in Demographic Group | p= (chi2 test) |
| Asian | 25.0% | 29.1% | 4 | 0.858 |
| Black | 50.0% | 28.7% | 8 | 0.188 |
| Native American | 20.0% | 29.1% | 5 | 0.655 |
| Multiple Race | 33.3% | 28.9% | 21 | 0.066 |
| White | 28.7% | 34.2% | 544 | 0.467 |
| BIPOC/Hispanic | 36.2% | 28.4% | 47 | 0.261 |
| Hispanic | 50.0% | 28.4% | 16 | 0.061 |

**1.3 Supplementary Table 3.** Percent of respondents making less than $50,000 annually that indicated they often or sometimes experienced aspects of food insecurity based on whether or not they used a food pantry since the start of the COVID-19 pandemic (n=259). P values determined through chi-square tests.

| Food insecurity experience | Used Food Pantry | No Food Pantry | p= |
| --- | --- | --- | --- |
| The food that my household bought just didn’t last, and I/we didn’t have money to get more. | 20.0% | 41.2% | 0.000 |
| I/we didn’t have money to get more | 20.2% | 40.7% | 0.000 |
| Adults in our household had to cut the size of your meals or skip meals because there wasn’t enough money for food. | 15.6% | 21.1% | 0.000 |
| I/we have eaten less that I/we felt I/we should because there wasn’t enough money for food | 17.0% | 20.8% | 0.000 |
| Adults in our household cut the size of our meals or skip meals because there wasn’t enough money for food | 17.1% | 21.3% | 0.000 |
| I/we were hungry but I/we didn’t eat because there wasn’t enough money for food. | 13.9% | 18.9% | 0.000 |

## 2.0 Supplementary Figures


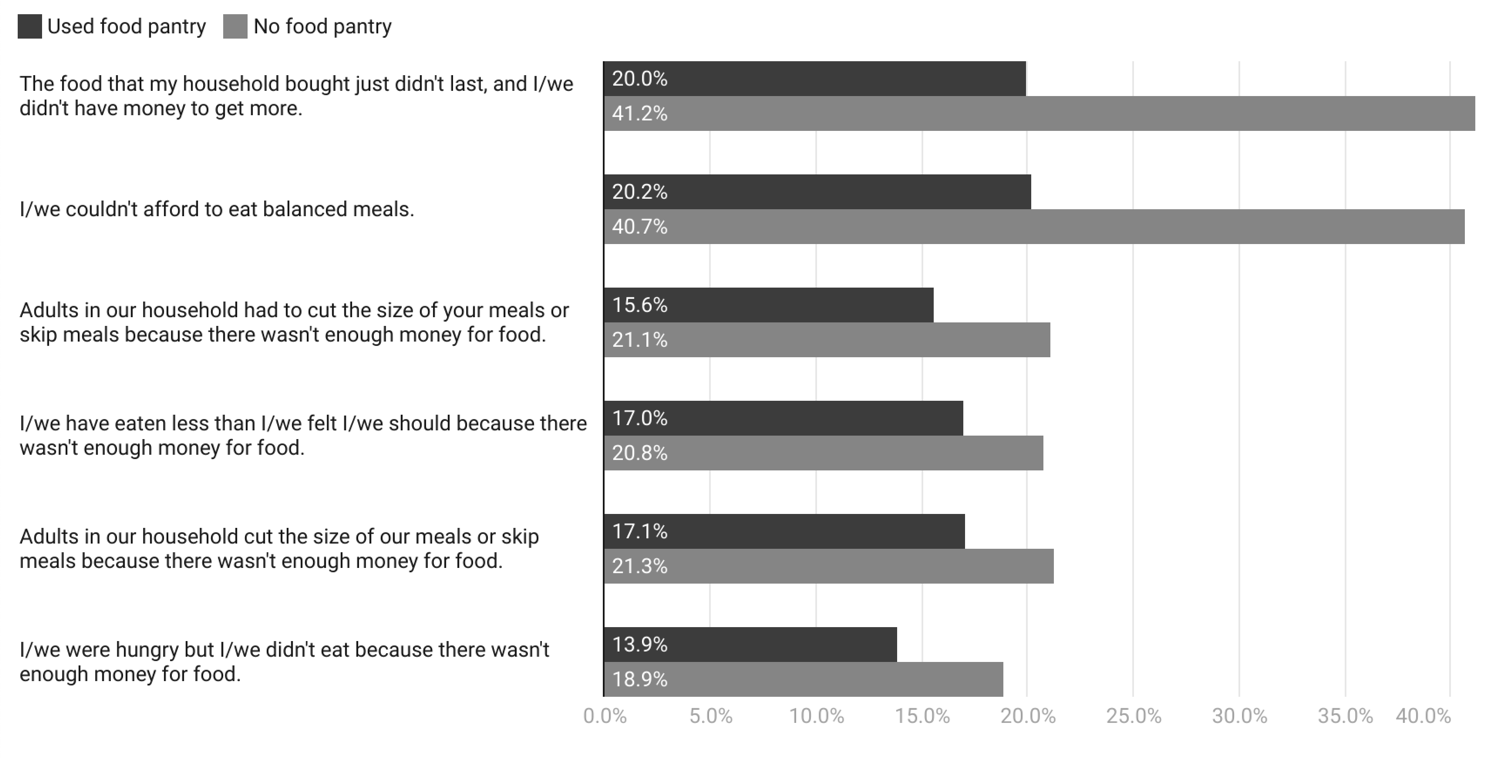


**2.1 Supplementary Figure 1:** Percent of respondents making less than $50,000 annually that indicated they often or sometimes experienced aspects of food insecurity based on whether or not they used a food pantry since the start of the COVID-19 pandemic (n=259). Chi-squared p-value <0.001 for all differences (Supplementary Table 4).


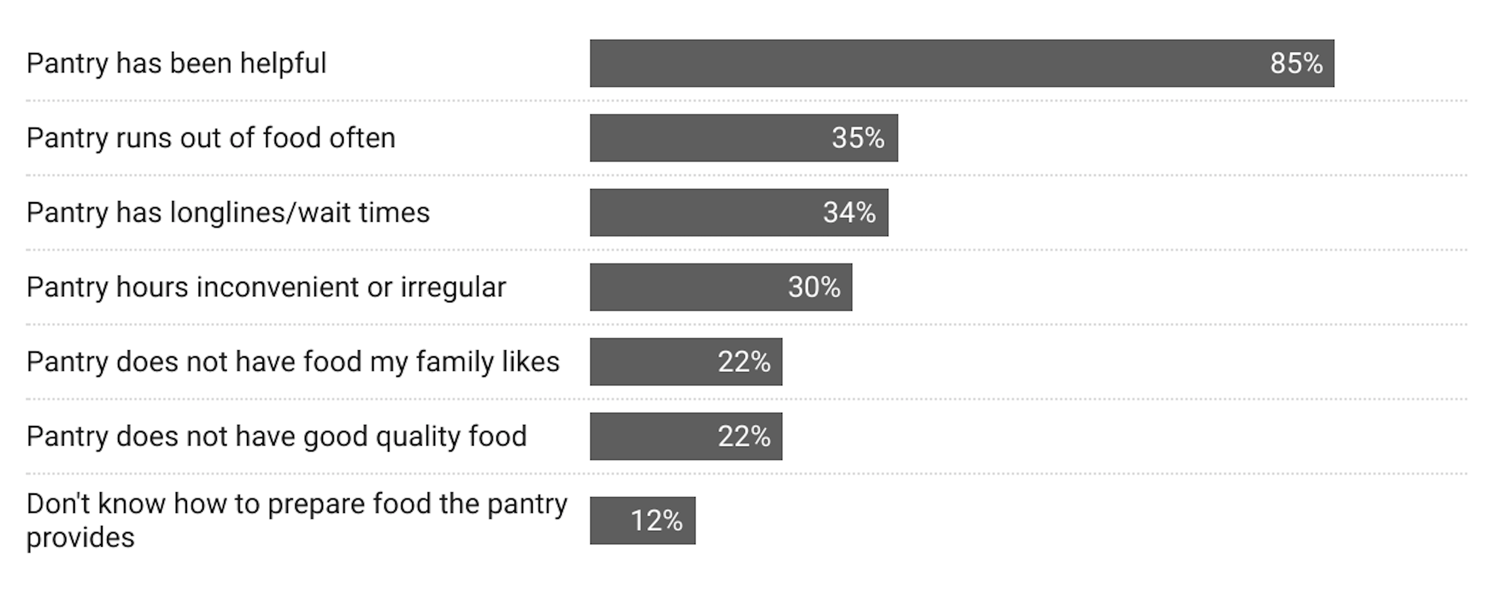


**2.2 Supplementary Figure 2.** Participant experiences related to using food pantries during the COVID-19 pandemic (N=86).

Footnote: Includes respondents who strongly agreed or agreed with the statement.
